# In Vitro Differentiation of Human Placenta-Derived Multipotent Cells into Schwann-Like Cells

**DOI:** 10.3390/biom10121657

**Published:** 2020-12-10

**Authors:** Chung-Hau Juan, Mei-Hsiu Chen, Feng-Hui Lin, Chih-Shung Wong, Chih-Cheng Chien, Ming-Hong Chen

**Affiliations:** 1 Department of Anesthesiology, Cathay General Hospital, Taipei 106438, Taiwan; chjuan@cgh.org.tw (C.-H.J.); w825567@gmail.com (C.-S.W.); chiencmail@gmail.com (C.-C.C.); 2 Department of Biomedical Sciences, National Central University, Taoyuan 32001, Taiwan; 3 Department of Internal Medicine, Far Eastern Memorial Hospital, New Taipei City 220216, Taiwan; michelle8989@gmail.com; 4 Department of Biomedical Engineering, Ming Chuan University, Taoyuan 333321, Taiwan; 5 Department of Biomedical Engineering, National Taiwan University, Taipei 106319, Taiwan; double@ntu.edu.tw; 6 Department of Neurosurgery, Taipei Municipal Wangfang Hospital, Taipei 116081, Taiwan; 7 Department of Biomedical Sciences, Graduate Institute of Nanomedicine and Medical Engineering, Taipei Medical University, Taipei 110301, Taiwan

**Keywords:** placenta-derived multipotent stem cell, differentiation, Schwann cell, peripheral nerve

## Abstract

Human placenta-derived multipotent stem cells (PDMCs) resembling embryonic stem cells can differentiate into three germ layer cells, including ectodermal lineage cells, such as neurons, astrocytes, and oligodendrocytes. The favorable characteristics of noninvasive cell harvesting include fewer ethical, religious, and legal considerations as well as accessible and limitless supply. Thus, PDMCs are attractive for cell-based therapy. The Schwann cell (SC) is the most common cell type used for tissue engineering such as nerve regeneration. However, the differentiation potential of human PDMCs into SCs has not been demonstrated until now. In this study, we evaluated the potential of PDMCs to differentiate into SC-like cells in a differentiation medium. After induction, PDMCs not only exhibited typical SC spindle-shaped morphology but also expressed SC markers, including S100, GFAP, p75, MBP, and Sox 10, as revealed by immunocytochemistry. Moreover, a reverse transcription-quantitative polymerase chain reaction analysis revealed the elevated gene expression of S100, GFAP, p75, MBP, Sox-10, and Krox-20 after SC induction. A neuroblastoma cell line, SH-SY5Y, was cultured in the conditioned medium (CM) collected from PDMC-differentiated SCs. The growth rate of the SH-SY5Y increased in the CM, indicating the function of PDMC-induced SCs. In conclusion, human PDMCs can be differentiated into SC-like cells and thus are an attractive alternative to SCs for cell-based therapy in the future.

## 1. Introduction

Peripheral nerve injuries are common clinical events that can have harmful outcomes including major disabilities that create an economic burden on society [1]. Most peripheral nerve defects are treated with direct end-to-end repair, nerve repair with autologous nerve grafts, or nerve conduits for large nerve defects. However, functional recovery remains poor despite optimal surgical repair [2]. A meta-analysis in 2005 of median and ulnar nerve repairs demonstrated that only 51.6% achieved satisfactory motor recovery and only 42.6% achieved sensory recovery [3]. Techniques involving tissue engineering and cell-based therapy are an alternative for nerve repair with Schwann cell (SC) transplantation [4]. SCs, which exist in the peripheral nervous system and cover nerve fiber axons, can produce neurotrophic factors, extracellular matrix molecules, and integrins, which provide trophic guidance and structural support for axon regeneration [5]. Moreover, SCs are central in peripheral nerve regeneration and are the most common cell type used in tissue engineering techniques. SCs are also essential in therapy for central nervous system (CNS) or demyelinating diseases, such as multiple sclerosis, spinal cord injury, or CNS injury [4,6,7,8]. However, using adult SCs have certain limitations; for example, they require invasive harvesting and sacrificing other functional nerves with consequent neurological deficits or neuroma formation, and allogeneic SCs have immune reactions [9]. On the other hand, stem cells can be used to acquire SCs through transdifferentiation methods. Mesenchymal stem cells (MSCs) are currently one of the promising sources for cell-based therapy. Some researchers have indicated that rat MSCs can differentiate into SC-like cells under certain conditions [10]. Human MSCs also exhibited the ability to differentiate into SC-like cells [11]. Compared with MSCs and stem cells from other sources, placenta-derived multipotent stem cells (PDMCs) have several advantages, including noninvasive harvesting and fewer ethical and legal concerns. PDMCs exhibit similar transdifferentiation and plasticity as do bone marrow MSCs under certain conditions [11]. The ability of PDMCs to differentiate into three layers of tissue, including bone, fat, or nerve tissue, renders them a promising source for cell-based therapy and tissue engineering [12,13,14]. However, the potential of PDMCs to differentiate into SCs remains to be demonstrated. This study evaluated the potential of PDMCs to differentiate into SC-like cells in an induction medium. To characterize PDMC differentiation, we examined the gene and protein expression of SC markers by using a reverse transcription-quantitative polymerase chain reaction (qRT-PCR) and immunofluorescence. Moreover, a functional assay of differentiated PDMCs was performed to evaluate whether soluble growth factors secreted from induced PDMCs facilitated the neurite outgrowth of neuroblastoma cells.

## 2. Materials and Methods

### 2.1. Isolation and Culture of Placenta-Derived Multipotent Stem Cells

After obtaining approval from the Institutional Review Board (CHIRB No. CT750) and written informed consent from mother, the placenta was collected after birth and sent to our laboratory forthwith. The amniotic membrane was removed, and the placental tissue was minced into small pieces. The sample was digested enzymatically, centrifuged, and seeded into an expansion medium consisting of Dulbecco’s modified Eagle’s medium (DMEM) (Hyclone, Thermo, MA, USA) with 10% fetal bovine serum (FBS) (SAFC Biosciences, KS, USA), 100 U/mL penicillin, and 100 μg/mL streptomycin (Gibco, Invitrogen, MA, USA), and incubated in a humidified 5% CO_2_ 95% air incubator at 37.5 °C. When the cells obtained more than 80% confluence, they were subjected to 1:2 subculture.

### 2.2. Flow Cytometry Analysis

To characterize cells cultured from the placenta, immunophenotyping expression was performed using FACS Caliber (BD Biosciences, CA, USA). Cells were trypsinized and labeled with fluorescein isothiocyanate (FITC)-conjugated antibodies, including anti-CD117, anti-CD34, anti-CD9, anti-CD44, anti-CD90, anti-CD45, anti-HLA-DR (BD Biosciences, CA, USA); anti-CD13, anti-CD 14, anti-CD29 (Biolegend, San Diego, CA, USA); anti-CD105, anti-CD49e, anti-CD54, anti-Stro-1 (Chemicon, Temecula, CA, USA); and anti-CD166 and anti-HLA-ABC (Serotec, Raleigh, NC, USA). Antibodies against SH3 and SH4 were obtained with the cell lines (BCRC, City, Taiwan). A secondary antibody was used with the FITC-conjugated anti-mouse IgG antibodies (BD Biosciences, CA, USA) when appropriate.

### 2.3. Induction of Differentiation into Schwann Cell-Like Cells

After passage 5, PDMCs were seeded on coverslips and cultured for 1 d in the expansion medium. The culture medium was then replaced with αMEM (Hyclone, Thermo) containing 10% FBS, 2 mM L-glutamine, 100 U/mL P/S, 35 ng/mL, and additional 1 mM β-mercaptoethanol (β-ME) (Merck, Calbiochem, CA, USA) and incubated for 24 h. After three washes of phosphate-buffered saline (PBS), the medium was replaced with 35 ng/mL of all-trans-retinoic acid (RA) (Sigma, MO, USA), and the cells were incubated for 72 h. A differentiation medium containing 10 μM forskolin (Merck, Calbiochem), 10 ng/mL of basic fibroblast growth factor (bFGF), 200 ng/mL of heregulin-beta (Her-β), and 5 ng/mL of PDGF-AA (all from Sigma) was used for the PDMCs; the cells were incubated in the medium for 14 d, and the medium was changed every 3 d. Morphological assessment, immunocytochemical studies, and reverse transcription-polymerase chain reaction (RT-PCR) were performed on days 5, 8, 11, and 14.

### 2.4. Quantitative Real-Time Polymerase Chain Reaction

On days 5, 8, 11, and 14 after PDMC induction, the RNA was isolated from the incubated PDMCs using a TRIzol Reagent (Invitrogen Life Technologies, Carlsbad, CA, USA) and reverse transcribed to cDNA. The Roche LightCycler with SYBR Green reagents (LightCycler-DNA Master SYBR Green I kit; Roche, Basel, Switzerland) was used throughout the RT-PCR process according to the manufacturer’s instructions. Table 1 lists the oligonucleotide primers used. The amplification program consisted of a preincubation phase at 95 °C for 10 min and an amplification phase of 30–50 cycles consisting of a denaturing phase at 96 °C for 15 s, an annealing phase at 55 °C for 10 s, and an elongation phase at 72 °C for 60 s. After the PCR, the results were collected and analyzed. Three independent samples were examined in each studied group.

### 2.5. Immunofluorescence Analysis

Cultured cells were fixed in 4% paraformaldehyde for 20 min at room temperature. After three PBS washes, 10% horse serum was added as a blocking agent. The samples were then incubated for 1 h at room temperature. After adding primary antibodies, the samples were incubated overnight at 4 °C. The samples were subsequently incubated with FITC-conjugated secondary antibodies for 2 h, followed by incubation with DAPI solution (3 μg/mL) for nucleic staining. For immunofluorescence staining, primary antibodies against S100 (1:200, Dako, CA, USA), Sox 10 (1:200, Santa Cruz Biotechnology, KS, USA), GFAP (1:100, Sigma, MO, USA), MBP (1:400, Sigma, MO, USA), p75 (1:100, Sigma, MO, USA), and MAP2 (1:100, Sigma, MO, USA) and fluorescein-labeled anti-rabbit secondary antibodies (1:500, Chemicon, IL, USA) were used. The cells were observed and photographed under a fluorescence microscope (Olympus BX61) by using Olympus Soft Imaging Solution software (analysis LS Research Ver. 2.7).

### 2.6. Conditioned Medium from Schwann Cell-Like Cells for SH-SY5Y Cell Culture

To evaluate whether SC-like cells can secrete soluble factors to promote the differentiation of SH-SY5Y cells, the cultured medium from induced PDMCs was collected and seeded with SH-SY5Y cells. Ten days after PDMC induction, the culture medium of PDMCs was replaced with DMEM containing 2% FBS, and the cells were incubated for two more days. The culture medium was collected and regarded as a conditioned medium (CM), which was subsequently used to culture SH-SY5Y cells. In the control group, SH-SY5Y cells were cultured with DMEM and 2% FBS. The morphology was examined under a phase contrast microscope after culturing for 2 d.

## 3. Results

### 3.1. Characterization of Human Placenta-Derived Multipotent Cells

**Figure 1:**
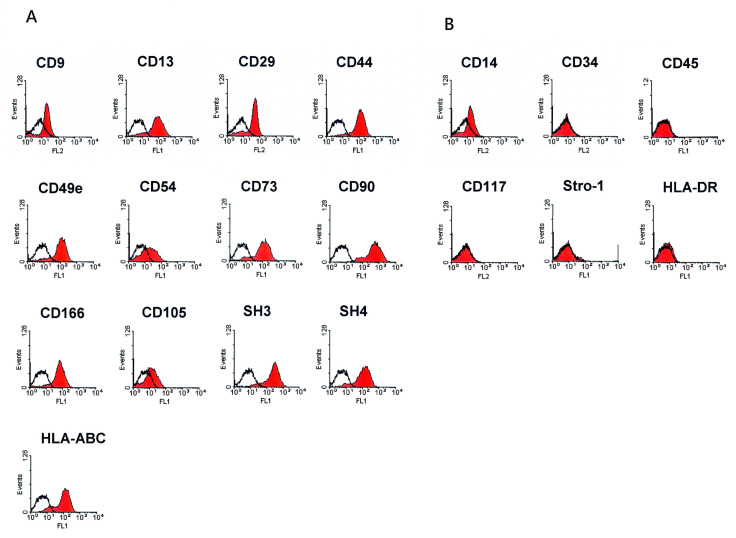
Phenotypic expression of placenta-derived multipotent stem cells (PDMCs). The results of flow cytometry revealed HLA-ABC^+^, CD29^+^, CD44^+^, CD9^+^, CD90^+^, CD105^+^, SH3^+^, SH4^+^, CD166^+^, CD13^+^, CD49e^+^, and CD54^+^ and HAL-DR^-^, CD45^-^, CD34^-^, Stro-1^-^, and CD117^-^. (**A**,**B**) markers for MSCs (mesenchymal stem cell) and hematopoietic cells, respectively.

The PDMCs were isolated from a human placenta, and flow cytometry was used to define the immunophenotypes of the isolated cells. The results were consistent with those of previous studies, whereby undifferentiated cells were positive for markers common to MSCs, including CD29, CD44, CD90, CD105, SH3, SH4, CD166, and CD9. These cells were also positive for the markers CD13, CD49e, CD54, and HLA-ABC but negative for the hematopoietic surface markers, CD34, CD45, CD117, HLA-DR, and Stro-1 (Figure 1).

### 3.2. Differentiation into Schwann Cell-Like Cells

**Figure 2:**
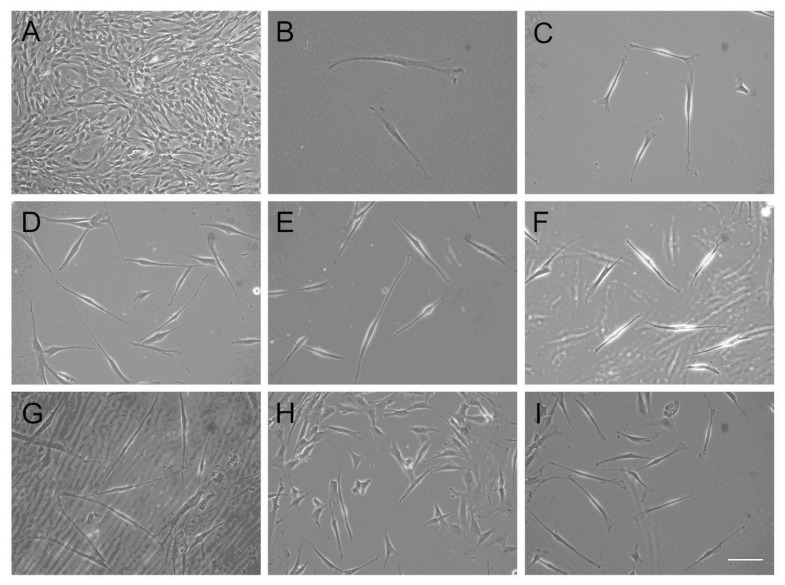
Morphological changes after Schwann cell (SC)-cell like cell induction. Undifferentiated human placenta-derived multipotent stem cells (PDMCs) at passage 4 exhibited fibroblast-like morphology under a phase contrast microscope (**A**). After incubation with β-ME, PDMCs began to elongate and left a contracted cell body with several process-like extensions (**B**). After incubation with RA, cells further retracted their cytoplasm to become spindle shaped and produced more obvious halations (**C**). After induction with neurotrophic and growth factors, human PDMCs differentiated into SC-like cells on days 5, 8, 11, and 14 (**D**, **E**, **F**, **G**, respectively). A higher percentage of morphological change was exhibited from days 5–8 (D, E). SC-like cells retained their morphology on day 11 (F). Some SC-like cells failed to maintain their morphology and even died (G). Relationship between morphology and cell density; morphological changes of cells were less obvious in areas with a higher cell density (**H**) than in those with a lower cell density (**I**). Bar, 100 μm.

To induce the differentiation of PDMCs into SC-like cells, PDMCs were incubated in the differentiation medium as described earlier. The morphology of the PDMCs was fibroblast-like (Figure 2A), similar to that of the control group. After incubation with β-ME, the cytoplasm of the large, flat PDMCs began to produce a light halation, leaving a contracted cell body with a few process-like extensions (Figure 2B). After incubation with RA, the differentiated cells exhibited a further retraction of the cell body and became spindle shaped (Figure 2C). After stimulation with a cocktail of growth factors, the induced SC-like cells elongated and had bipolar processes. Their morphology became gradually similar to that of human SCs with an increasing induction period on days 5, 8, 11, and 14 (Figure 2D–G). The morphological changes of the induced PDMCs could rarely be observed before the third day; however, they became more noticeable after 5 d of differentiation induction. During the induction period, we noticed that the morphological changes were related to the density of cells seeded. Changes in the morphology of typical SCs were more obvious in areas with a lower cell density (Figure 2H,I). In the less confluent layer, the morphology of approximately one-third to one-half of the cells adopted a typical SC morphology (Figure 2I).

### 3.3. Characterization of Differentiated Placenta-Derived Multipotent Stem Cells

**Figure 3:**
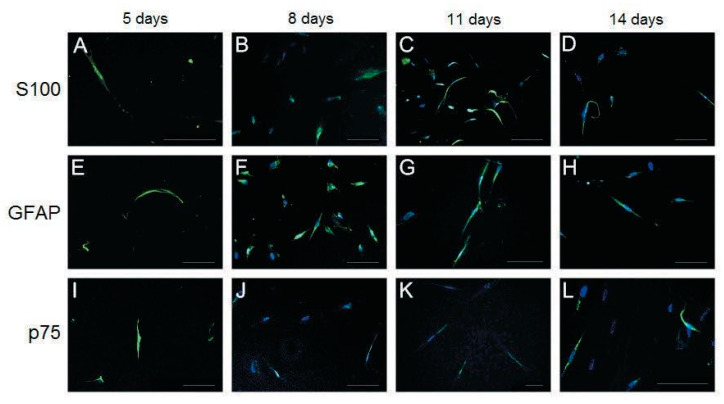
Immunofluorescence staining of differentiated placenta-derived multipotent stem cells (PDMCs) after culturing in the induction medium from Schwann cell-like cells for 5, 8, 11, and 14 days. Expression of S100 (**A**–**D**), GFAP (**E**–**H**), and p75 (**I**–**L**) was observed from days 5 to 14; however, the highest expression was exhibited on day 11 (**C**,**G**,**K**). Nuclei are labeled with DAPI (4′,6-diamidino-2-phenylindole) (blue). Bar, 100 μm.

**Figure 4:**
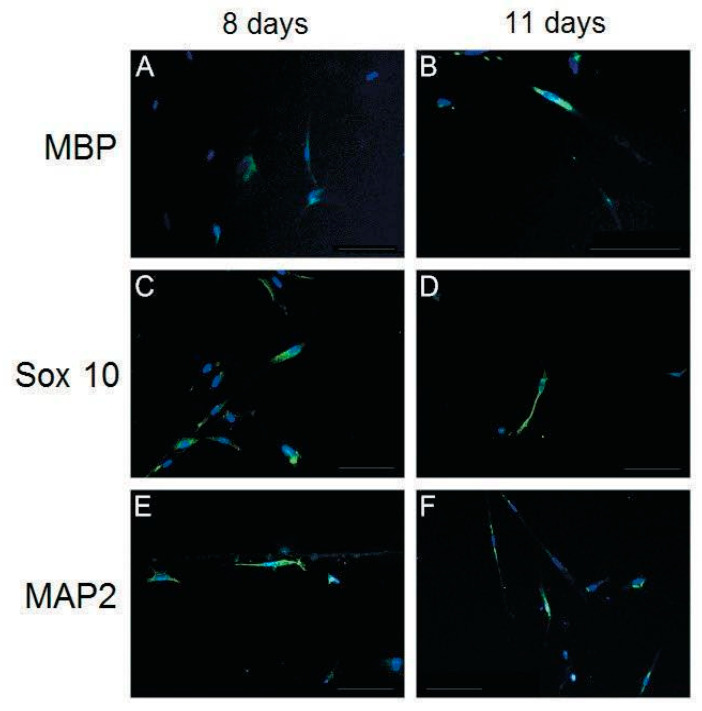
Characterization of differentiated placenta-derived multipotent stem cells (PDMCs) after culturing with the induction medium from Schwann cell-like cells for 8 and 11 days. Expression of MBP (myelin basic protein) and Sox 10 was observed on days 8 and 11 (**A**–**D**) but not on days 5 or 14 (data not shown). The highest expression of MBP was observed on day 11 (**B**). By contrast, the highest expression of Sox 10 was observed on day 8 (C). Immunofluorescence staining of MAP2 (Microtubule-associated protein 2) was slightly higher on day 8 (**E**) than on day 11 (**F**). Nuclei are labeled with DAPI (4′,6-diamidino-2-phenylindole) (blue). Bar, 100 μm.

In addition to morphological changes, the expression of SC markers, including S100, glial fibrillary acidic protein (GFAP), low-affinity nerve growth factor receptor (p75), myelin basic protein (MBP), Transcription factor SOX-10 (Sox 10), and the neuronal marker microtubule-associated protein 2 (MAP2) was investigated using immunofluorescence (Figure 3 and Figure 4).

S100, GFAP, and p75 were expressed through days 5–14; however, their expression was highest on day 11 (Figure 3C,G,K). The protein expression of MBP, Sox 10, and MAP2 was observed only on days 8 and 11 in our study model (Figure 4). The highest expression of MBP was observed on day 11 (Figure 4B), and the highest expression of Sox 10 was observed on day 8 (Figure 4C). The expression of MAP2, a marker of mature neurons, was observed on days 8 and 11 (Figure 4E,F), with a slightly higher expression on day 8.

**Figure 5:**
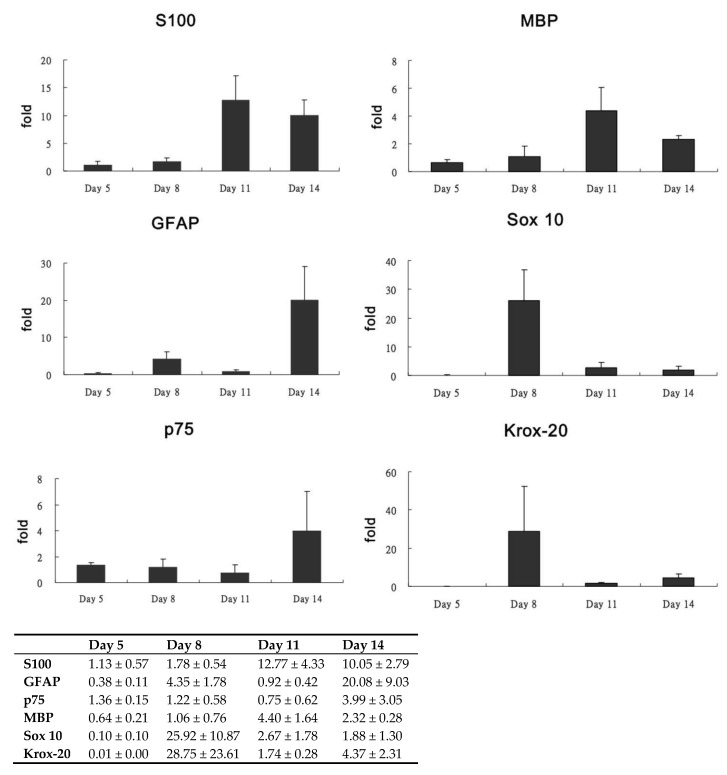
Ratio of mRNA expression of various genes to that of GAPDH (Glyceraldehyde 3-phosphate dehydrogenase) on days 5, 8, 11, and 14. Expression of S100 and MBP (myelin basic protein) was observed markedly on days 11 and 14. The highest expression of GFAP (Glial fibrillary acidic protein) and p75 was observed on day 14. Comparatively, the early developmental markers of Schwann cells, Sox 10 and Krox-20, were most noticeable on day 8.

Various mRNAs were also examined for gene expression (Figure 5). The mRNA expression of S100 and MBP was noted on days 11 and 14, with the most remarkable expression on day 11, consistent with the immunofluorescence analysis results. The highest mRNA expression of GFAP and p75 was observed on day 14. The early developmental markers of SCs, such as Sox 10 and early growth response protein 2 (Krox-20/Egr2), were most noticeable on day 8.

### 3.4. Culture of SH-SY5Y Cells in the Conditioned Medium from Differentiated Placenta-Derived Multipotent Stem Cells

**Figure 6:**
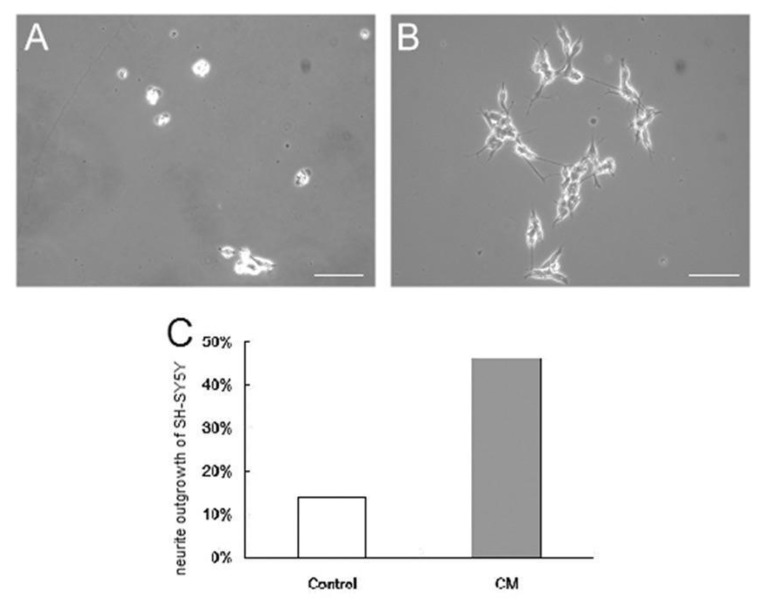
SH-SY5Y cells cultured with the conditioned medium from differentiated placenta-derived multipotent stem cells (PDMCs). The conditioned medium (CM) from Schwann cell-like cells promoted more neurite sprouting from the SH-SY5Y cells (**B**) than did the control group (**A**). The longest length and average length of neurites were also greater in the CM group (**B**). The neurite outgrowth of SH-SY5Y cells was 14.03% in the control group and 46.38% in the CM group (**C**). Bar, 100 μm.

To evaluate whether induced SC-like cells could secrete soluble factors that could promote the differentiation of SH-SY5Y cells, a human neuroblastoma cell line, the cultured medium from induced SC-like cells was collected and seeded with SH-SY5Y cells. After culturing in the CM collected from SC-like cells for 2 days, approximately one-half of the SH-SY5Y cells exhibited neurite outgrowth under a phase contrast microscope. Neurite positive cells were defined as those cells extending at least one neurite greater than one cell-body diameter [15]. However, the control group exhibited substantially less neurite outgrowth. The neurite outgrowth of SH-SY5Y cells was 46% in the CM group compared with 14% in the control group under the phase contrast microscope (Figure 6). The average neurite and the longest neurite in the CM group were also apparently longer than those in the control group, indicating the differentiation potential of the CM.

## 4. Discussion

Stem cells are a crucial source of cells for tissue engineering and regenerative medicine. The advantages of PDMCs, including a promising potential, noninvasive harvesting, unlimited sources, and fewer legal and ethical concerns compared with those of other stem cells, have been widely discussed [14]. PDMCs have exhibited the ability to differentiate into several types of cells, including adipocyte, osteoblast, hepatocyte, and neural lineage cells (neurons, glial cells, and oligodendrocytes) [13,16,17]. However, the ability of human PDMCs to differentiate into SCs has not been demonstrated. Previous studies have revealed that human MSCs can differentiate into SC-like cells [9,11]; because PDMCs have characteristics similar to those of MSCs, we tested the ability of PDMCs to differentiate into SC-like cells.

Previous studies have indicated that MSCs can potentially differentiate into SC-like cells. Incubation with β-ME for 1 d can possibly promote the differentiation of human stromal cells into the neural lineage [18]. RA is also central in inducing the differentiation of mouse embryonic stem cells into the neural lineage [19]. Other reagents, including forskolin, bFGF, Her-β (a subgroup of neuregulin [NRG]), and PDGF-AA were used to induce the differentiation of MSCs into SC-like cells [9]. NRGs play an essential role during gliogenesis and can affect the outcome of boundary cap neural crest stem cells in maturing into myelinating SCs under certain environmental conditions [20]. The survival and development of SCs were regulated by signals, including β-NRGs, insulin-like growth factors, platelet-derived growth factor (PDGF), and neurotrophin 3 [21,22]. Forskolin, acting as an activator of adenylyl cyclase, synergistically promotes other growth factors, including bFGF, IGF, and PDGF, during SC growth [23]. The activation of the mitogen-activated protein kinases ERK1 and ERK2 and the autophosphorylation of the Her receptors erbB2 and erbB3 in SCs were stimulated by the epidermal growth factor homology domain of β-Her [24]. In this study, we isolated and cultured PDMCs from properly treated and minced human placental tissues. The PDMCs we obtained had typical characteristic immunophenotyping expressions which were positive for markers common to MSCs and negative for the hematopoietic surface markers (Figure 1). After induction, the undifferentiated PDMCs (Figure 2A) differentiated into morphological SC-like cells gradually by time (Figure 2B–F). We noticed that the morphological changes were the density of cells seeded. Changes in the morphology were more obvious in areas with lower cell density (Figure 2H,I). The cell seeding density affected the proliferation and differentiation of the stem cells has been explored [25]. We believe over-loaded cells face contact-inhibition, limited nutrients, hypoxia, and insufficient waste removal. If the cell density is adequate, cell–cell interactions and intercellular signal molecules may stimulate cells producing more extracellular matrix to enhance each other’s viability and functions.

Because no previous studies have reported the differentiation potential of human PDMCs into SC-like cells, we used various phenotypic markers to identify the differentiation of PDMCs into mature SC-like cells; these included S100, GFAP, p75, MBP, Sox 10, and Krox-20/Egr-2, which are expressed both at the transcriptional and translational levels (Figure 3, Figure 4 and Figure 5). The highest expression of all these markers was observed on day 11. Transcription factors, such as Sox 10 and Krox-20, are required during myelination and have been used as markers for identifying SCs in recent studies [26,27]. In our model, a substantial gene expression of Sox 10 and Krox-20 on day 8 after induction also indicated the transdifferentiation of PDMCs into the SC-like cells, consistent with the results of previous studies. MBP exists only in myelinating cells, such as myelinating SCs or oligodendroglia [28]. The expression of MBP in our study suggested that SC-like cells differentiated from PDMCs could myelinate. Approximately 5% of differentiated rat MSCs have reportedly exhibited morphological changes to other neural lineages, such as astrocytes, oligodendrocytes, and neurons [9]. According to the analysis of the inducing reagents we used, the possibility of the induction of neural lineage cells was low. In addition to glial markers, we observed the expression of MAP2 in differentiated PDMCs. MAP2 is a marker for mature neurons that belongs to a family of microtubule-associated proteins and is crucial during neural development [29,30]. Furthermore, we found a similar result that indicated an increased cell death rate after transdifferentiation, particularly after 14 d of differentiation (Figure 2G). However, we believed a live/dead assay could provide more insight into the death rate occurring in the course of the differentiation setting. This would be also useful for estimating the efficiency and potentiality of the SC differentiation protocol. According to studies performed on the SC differentiation of various types of stem cells, the duration of induction may vary from 8 to 14 days [9,31,32]. Immunocytochemistry and flow cytometry indicated that the expression of the S100 protein in differentiated MSCs differed at various time points after induction [31,32,33]. Comparatively, in our study model, the expression of S100 was first detected on day 5 and reached the highest level on day 11 after induction. The peak mRNA expression of Sox 10 and Krox-20 in our study was observed simultaneously on day 8. Krox-20 is a transcription factor responsible for myelination and is only expressed in myelinating SCs [6]. Sox 10 is a key regulator in early glial development [34,35]. The expression of Sox 10 and Krox-20 revealed that PDMCs began to develop into SC-like cells 8 days after induction. Thus, the phenotypic characteristics of SCs, as indicated by the expression of SC markers, including S100, GFAP, MBP, and p75, on days 11 and 14, suggested that the PDMCs were induced into SC-like cells.

Although immunofluorescence and RT-PCR results indicated that PDMCs differentiated into SC-like cells, they did not imply that the SC-like cells had the same biological functions as did mature SCs. The secretion of growth factors by functional SCs can contribute to the nerve development mechanism and axonal regeneration [36,37]. The projection from the cell body of a neuron, also known as the neurite, was seen at developing neurons. SC-conditioned medium could induce neurite outgrowth in SH-SY5Y neuroblastoma cell culture [36]. Using our SC-like cells conditioned medium collected on day 10 (because of the highest expression of SC makers, such as S100, GFAP, and MBP) successfully induced the neurite outgrowth in a neuroblastoma cell line, SH-SY5Y cells (Figure 6). In this study, we proved our SC-like cells differentiated from PDMCs were functioning Schwann cells by using their culture medium to induce axonal growth in a neuroblastoma cell line, SH-SY5Y cells. The SC-like cells differentiated from human PDMCs not only possessed similar morphological and phenotypic characteristics, and, more importantly, similar functions like those of normal SCs.

## 5. Conclusions

The results of this study demonstrated that human PDMCs could differentiate into cells with both morphological and phenotypic characteristics of the SC lineage. Furthermore, SC-like cells differentiated from PDMCs exhibited functional potential and could enhance neurite outgrowth in vitro in the conditioned medium bioassay. Hence, human PDMCs can be a potential source of SC substitutes for cell-based therapy in the future.

## Figures and Tables

**Table 1:** Primer sequences for RT-PCR.

Gene	Forward Primer (5′-3′)	Reverse Primer (5′-3′)
S100β	AGACCAGGAAGGGGTGAGA	CCTCCCTTCCAGAATATTGGT
GFAP	GAAGGTTGAGTCGCTGGAGGAG	CGCTGTGAGGTCTGGCTTGG
p75	CCAGCAGACCCATACGCAGAC	GCCAGATGTCGCCAGGTATCC
MBP	GCACAGAGACACGGGCATCC	CGGGCATGAGAAGGCAGAGG
Sox 10	AACGGCGCCAGCAAAAGCAA	CGGGCATGAGAAGGCAGAGG
Krox-20	CTCACGCCACTCTCCACCATC	CCTCACCGCCTCCACTTGC
GAPDH	ATGATTCTACCCACGGCAAG	CTGGAAGATGGTGATGGGTT

S100β: S100 protein, β polypeptide; GFAP: glial fibrillary acidic protein; p75: low affinity nerve growth factor receptor; MBP: myelin basic protein; Sox 10: transcription factor; Krox-20 = Egr2: early growth response protein 2; GAPDH: glyceraldehyde-3-phosphate dehydrogenase.
